# A recombinant antigen-based enzyme-linked immunosorbent assay (ELISA) for lungworm detection in seals

**DOI:** 10.1186/s13071-015-1054-4

**Published:** 2015-09-02

**Authors:** Sophia Arlena Ulrich, Kristina Lehnert, Ursula Siebert, Christina Strube

**Affiliations:** Institute for Terrestrial and Aquatic Wildlife Research, University of Veterinary Medicine Hannover, Werftstrasse 6, 25761 Buesum, Germany; Institute for Parasitology, University of Veterinary Medicine Hannover, Buenteweg 17, 30559 Hannover, Germany

**Keywords:** ELISA, Major sperm protein, *Phoca vitulina*, *Halichoerus grypus*, Lungworm infection, Verminous pneumonia, Metastrongyloidea, *Otostrongylus circumlitus*, *Parafilaroides gymnurus*

## Abstract

**Background:**

Pinnipeds are frequently infected by the lungworms *Otostrongylus circumlitus* and *Parafilaroides gymnurus* (Metastrongyloidea). Infections are frequently associated with secondary bacterial bronchopneumonia and are often lethal. To date, a reliable lungworm diagnosis in individual seals is only possible during necropsy as examination of faeces collected from resting places does not allow assignment to individuals. Therefore, a diagnostic tool for lungworm detection in living seals is desirable for monitoring health of seals in the wild and in captivity. Previously, an ELISA based on recombinant bovine lungworm major sperm protein (MSP) as diagnostic antigen was developed for lungworm diagnosis in cattle. In the present study, this test was adapted for detection of antibodies against lungworms in harbour (*Phoca vitulina*) and grey seals (*Halichoerus grypus*). Furthermore, sera of northern elephant seals (*Mirounga angustirostris*) were tested to evaluate whether the harbour/grey seal ELISA is suitable for this seal species as well.

**Methods:**

For ELISA evaluation, lungworm-positive and -negative sera of harbour and grey seals were analysed using horseradish peroxidase (HRP)-conjugated Protein A as secondary antibody. Optical density was measured and a receiver operating characteristic (ROC) analysis was performed to determine a cut-off value. Potential cross-reactions were examined by testing serum of seals positive for gastrointestinal and heart nematodes, but negative for lungworm infections. In addition, sera of northern elephant seals were analysed.

**Results:**

Harbour and grey seal serum samples showed significant differences in optical density (OD) between serum of infected and uninfected animals resulting in a cut-off value of 0.422 OD with a specificity of 100 % (95 % CI: 87.23-100 %) and a sensitivity of 97.83 % (95 % CI: 88.47-99.94 %). Cross-reactions with heart or gastrointestinal nematodes were not observed. Analysis of northern elephant seal samples resulted in detection of antibodies in animals positive for lungworm larvae at faecal examination.

**Conclusions:**

The ELISA presented is a valuable method for detection of lungworm infections in live harbour and grey seals, providing a monitoring tool to reveal epidemiological dynamics of lungworm infections during health surveillance in free-ranging seals. Furthermore, ELISA results may aid institutions with harbour and grey seals under human care on decisions regarding anthelminthic treatment of individual animals.

## Background

Harbour seals (*Phoca vitulina*) and grey seals (*Halichoerus grypus*) are, beside harbour porpoises, the only marine mammals that reproduce in the German North and Baltic Sea [1–3]. They are frequently infected with the metastrongyloid nematodes *Otostrongylus circumlitus* and *Parafilaroides gymnurus* [4, 5]. These lungworms exhibit a high prevalence and pathogenicity and may cause obstruction of airways which is often accompanied by bacterial infections leading to severe bronchopneumonia and death [6–11].

Prevalence of lungworm infection in harbour seals, found in the German part of the North and Baltic Sea, ranged from 26 % in 1988/89 [12] and 76 % in 1997 to 2000 [8]. In grey seals, lungworms are less prevalent with generally lower intensity of infection than in harbour seals [4, 13]. Clinical symptoms of lungworm infection in harbour seals include emaciation, coughing, bronchial and nasal blood-speckled mucus as well as change of buoyancy conditions [14]. Adult harbour seals are rarely infected with lungworms and show a milder parasite intensity compared to individuals between two and 18 months of age [8]. Presumably, infection is acquired after weaning at approximately four weeks of age when young harbour seals start to prey on flatfish (e.g., dab, plaice, sole flounder or turbot) which are one of their main prey [15, 16]. Even though the life cycle of *O. circumlitus* and *P. gymnurus* is not fully described yet, benthic fish are potential intermediate hosts for marine lungworms [17–19]. It is also unknown if seals develop a protective immunity as it has been described for ruminants after infection with lungworms of the genus *Dictyocaulus* [20–24].

Lungworm infections in pinnipeds are also described in pacific seals. Northern elephant seals (*Mirounga angustirostris*) native on the channel islands of California, USA, and Baja California, Mexico, exclusively show infections with *O. circumlitus* [25, 7, 11]*.* Their adaptation to the parasite seems less pronounced than that of harbour or grey seals as mortality in northern elephant seals often occurs during the prepatent period of the lungworm infection [7, 26, 27]. Additionally, severity of pulmonary disease in northern elephant seals may not be correlated to intensity of infection [7].

To date, individual lungworm diagnosis for seals was solely conducted *post mortem* or by employing the Baermann technique [28] for detection of lungworm larvae, as commonly used for diagnosing lungworm infection in domestic and wild mammals. In parasitological studies involving seals, faecal collection is logistically challenging, as well as the assignment of samples to individuals. Additionally, the sensitivity of the methods is rather limited. Therefore, a reliable diagnostic tool for lungworm detection is required to evaluate exposure of lungworms in living seals. For immunodiagnosis of the bovine lungworm *Dictyocaulus viviparus*, an enzyme-linked immunosorbent assay (ELISA) detecting serum antibodies has been developed [29, 30]. Recombinant major sperm protein (MSP) was selected as antigen as it represents a protein family occurring in nematode sperm only. Thus, potential cross-reactions with trematode, cestode and acanthocephala infections are not to be expected. However, through its nature as sperm protein it is exclusively expressed by adult male worms [31, 32], therefore diagnosis is mainly restricted to patent infections. Because of the high sensitivity and specificity of each 100 % [30] of this diagnostic assay in detecting exposure to lungworms in cattle, it was aimed to adapt the ELISA to harbour and grey seals and to test its potential for diagnosing lungworms in northern elephant seals.

## Methods

### Sera of harbour and grey seals

To adapt and evaluate the ELISA for seals, serum samples of lungworm-positive and lungworm-negative seals were collected. Positive samples were obtained from approximately two to nine months old, free-living harbour and grey seals originating from the German North and Baltic coast. These animals were found in (very) poor health condition with infaust prognosis and thus were mercy killed by officially appointed seal rangers. Subsequently, seals were brought to the Institute for Terrestrial and Aquatic Wildlife Research (ITAW), University of Veterinary Medicine Hannover, for necropsy. Blood was taken with a 1.20 x 100 mm needle (SUPRA, Ehrhardt Medizinprodukte) either from the extradural intervertebral sinus 5 cm cranial to the pelvis [33] or by heart puncture. Blood was collected in serum separator tubes (S-Monovette®, Sarstedt) and subsequently centrifuged at 3000 x *g* for 15 min. Obtained serum was stored at −20 °C until use. Additionally, serum samples taken for routine diagnostic examinations from harbour and grey seals prior to rehabilitation were obtained from the Seal Sanctuary Friedrichskoog, Germany. Serum samples were assigned as lungworm-positive if (1) *O. circumlitus* or *P. gymnurus* were detected in lungs, heart or pulmonary artery during necropsy, or (2) lungworms were detected in histopathological lung sections or (3) lungworms were detected in sputum of living seals. Parasite species were determined by stereomicroscopic examination (45x magnification; Olympus SZ 61).

Negative serum samples taken for routine diagnostic examinations were obtained from zoos and animal parks. Sera included in ELISA evaluation originated from harbour and grey seals born and raised in human care without contact to their natural environment and fed on thawed fish only. Therefore, contact with lungworm antigens can almost certainly be excluded.

Finally, 46 samples of lungworm-positive individuals (45 harbour seals and one grey seal) and 27 samples of lungworm-negative individuals (20 harbour seals and seven grey seals) were available for adaption and evaluation of the MSP ELISA to harbour and grey seals.

### ELISA adaption to harbour and grey seals

#### Sera and conjugate dilutions

The ELISA for detection of antibodies against lungworms utilises recombinantly expressed MSP fused with *Schistosoma japonicum* glutathione-S-transferase (GST-MSP) as antigen. Recombinant antigen production and purification were performed as described previously [30]. Protein concentration was determined using Agilent 2001 Bioanalyzer (Agilent Technologies). Nunc® Immobilizer™ Amino-plates were coated with 0.25 μg GST-MSP/well diluted in 20 mM phosphate-buffered 150 mM saline (PBS, pH 7.4). The total volume per well was 100 μL. Plates were incubated overnight at 4 °C, washed three times for 5 min with PBS containing 0.05 % Tween-20™ (PBS-Tween), and tapped dry afterwards. Positive and negative harbour, as well as grey seal sera, were diluted 1:40 and 1:100, respectively, in PBS-Tween and 100 μL was added to the wells. Additionally, lungworm-positive and -negative cattle control sera were diluted 1:40 in PBS-Tween as previously described [30]. All samples were tested in duplicate. Plates were incubated for 1 h at 37 °C and then washed and tapped dry as described above. Recombinant horseradish peroxidase (HRP)-conjugated Protein A (Pierce®), capable of binding IgG, especially of carnivores, was used as secondary antibody in dilutions of 1:5,000, 1:10,000, 1:50,000 and 1:100,000 in PBS-Tween. Again, 100 μL were added to each well followed by incubation for 1 h at 37 °C. Before substrate application, plates were washed again followed by addition of 50 μL/well of σ-phenylenediamine dihydrochloride (0.4 mg/ml, Sigma-Aldrich) in 25 mM citrate/50 mM phosphate buffer comprising 0.04 % of a 30 % hydrogen peroxide solution. Incubation was carried out for 10 min in the dark at room temperature. The enzymatic reaction was stopped by adding 50 μL/well of 2.5 M sulphuric acid. Optical density (OD) was measured at a wavelength of 490 nm with the ELx800 ELISA Reader (Bio-Tek).

To select the most appropriate serum and conjugate dilution, OD values of two negative serum samples in dilutions of 1:40 and 1:100 in combination with four different Protein A (Pierce®) dilutions were subtracted from OD values of three positive serum counterpart samples. The dilutions resulting in the highest OD difference values were determined as conditions for future ELISA experiments.

### Analysis of pre-determined positive and negative serum samples

The pre-determined positive and negative serum samples of harbour and grey seals were analysed in duplicate using the final ELISA protocol, which includes serum sample dilution of 1:100 in PBS-Tween and HRP-conjugated Protein A (Pierce®) dilution of 1:50,000 in PBS-Tween. The arithmetic mean OD of the duplicates was calculated and corrected for the blank arithmetic mean OD.

### ELISA test parameter and cross-reactions

For cut-off determination and calculation of associated sensitivity and specificity, a receiver operating characteristic (ROC) analysis was carried out using MedCalc® (vol. 15.4, MedCalc Software). The curve was created by plotting the true positive rate (specificity) against the false positive rate (sensitivity) at various threshold settings to select a cut-off with the highest sensitivity and specificity.

To examine lungworm ELISA cross-reactions with heart and gastrointestinal nematodes, serum samples (*n* = 4) of harbour seals positive for microfilaria of the seal heartworm *Acanthocheilonema spirocauda* (Filarioidea) were examined in the ELISA with the protocol described above. Cross-reactions with gastrointestinal nematodes were tested by analysing serum samples (*n* = 6) of harbour seals showing infections with *Pseudoterranova decipiens* and *Contracaecum osculatum* (Ascaridoidea) during necropsy*.*

### Testing the lungworm ELISA with northern elephant seal sera

To test whether the harbour/grey seal-adapted ELISA is suitable for diagnosing lungworms in northern elephant seals (*Mirounga angustirostris*), 43 serum samples of the latter were obtained from The Marine Mammal Centre (TMMC) in Sausalito, California, USA. Samples originated from seals that were stranded alive at an age of less than one year on the coast of California, USA, and were brought to TMMC for rehabilitation. Serum samples were assigned as positive (*n* = 27) if *O. circumlitus* was detected in the lung, heart or pulmonary artery during necropsy. Additionally, faecal shedding of larvae was examined. If clinical symptoms of lungworm infection were lacking and simultaneously no lungworms were detected during necropsy, blood samples of the respective animals were considered as negative (*n* = 16). ELISA examinations were performed as described above.

### Immunoblotting

To visualise binding of seal anti-lungworm serum antibodies to recombinant bovine lungworm MSP, SDS-PAGE and subsequent immunoblotting were performed. Recombinant *D. viviparus* MSP was loaded on a 15 % gel as a mixture of GST-MSP fusion protein with pure MSP and pure GST, which were derived from thrombin cleavage of the fusion protein. After separation at 150 V for 70 min, proteins were transferred for 1 h at 100 mA onto nitrocellulose membranes (Porablot NCL, Macherey-Nagel) using the horizontal semi-dry technique. After rinsing the blots with Tris-buffered saline (TBS pH 7.8) for 5 min, the membranes were cut into strips and blocked with Roti®-Block (Carl Roth, Karlsruhe, Germany) in distilled water for 1 h at room temperature or overnight at 4 °C. After washing three times for 5 min with TBS containing 0.05 % Tween-20™ (TBS-Tween), the blots were incubated for 1 h at room temperature with defined positive and negative serum samples of harbour seals, grey seals and northern elephant seals, diluted 1:100 in TBS-Tween. The positive northern elephant seal sera used were the two that had reacted positive in previous ELISA experiments (cf. relevant results section). Additionally, ELISA-negative serum samples of northern elephant seals, which were lungworm-positive at necropsy as well as ELISA-negative samples of necropsy-negative animals were tested. After incubation with serum, membranes were washed as described above and incubated for 1 h with Protein A conjugated to alkaline phosphatase (AP) (Calbiochem®) diluted 1:5000 in TBS-Tween. Subsequently, membranes were washed twice for 5 min in TBS-Tween and once for 5 min in TBS. Antibody binding to MSP and/or GST was visualised through the AP-specific substrate BCIP (5-bromo-4-chloro-3-indolyl phosphate dipotassium)/NBT (nitrotetrazolium blue chloride).

## Results

### ELISA adaption to harbour and grey seals

#### Evaluation of serum and secondary antibody dilution

The assays showed the best discrimination between lungworm-positive and -negative samples using 1:100 diluted sera in combination with 1:50,000 diluted HRP-conjugated Protein A (Pierce®) as conjugate. These conditions were inserted in the final ELISA protocol.

### Analyses of pre-determined positive and negative serum samples

**Fig. 1:**
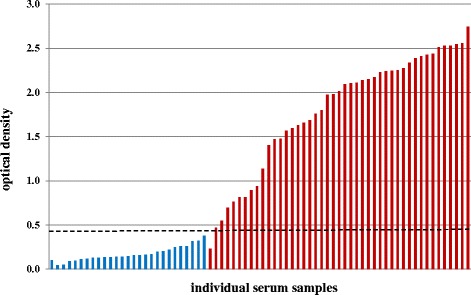
OD value distribution of harbour and grey seals. Lungworm ELISA OD distribution of lungworm-negative serum samples (blue bars) and lungworm-positive serum samples (red bars). The black dashed line marks the cut-off value of 0.422 OD

ODs between positive and negative serum samples were significantly different (t = 15.95, df = 47, *p* = <0.0001). Negative samples ranged from 0.044 OD to 0.376 OD, positive samples from 0.228 to 2.742 OD. The arithmetic mean of negative samples was 0.170 OD [standard deviation, (SD): 0.082] and of positive samples 1.794 OD (SD: 0.670). Figure 1 shows OD value distribution of serum samples graphically.

### ELISA test parameter and cross-reactions

Based on pre-determined lungworm-positive and -negative serum samples from harbour and grey seals, ROC analysis revealed a cut-off value of 0.422 OD with a specificity of 100 % (95 % CI: 87.23-100 %) and a sensitivity of 97.83 % (95 % CI: 88.47-99.94 %).

No cross-reactions were found with sera of harbour seals infected with heart nematodes as those resulted in OD values between 0.121 and 0.325 (arithmetic mean: 0.188 OD; SD: 0.094). Harbour seals infected with gastrointestinal nematodes revealed ODs between 0.161 and 0.322 (arithmetic mean: 0.233 OD; SD: 0.063 OD).

### ELISA examination of northern elephant seal samples

Negative serum samples of northern elephant seals resulted in ODs varying between 0.011 and 0.303 (arithmetic mean: 0.141; SD: 0.089). Twenty-five of the total 27 lungworm-positive samples resulted in OD values ranging between 0.028 and 0.235 OD (arithmetic mean: 0.096; SD: 0.058). These 25 animals did not show faecal excretion of lungworm larvae. The two remaining lungworm-positive northern elephant seal samples, originating from animals positive for faecal larvae shedding, resulted in OD values of 0.529 and 0.774.

### Immunoblotting

**Fig. 2:**
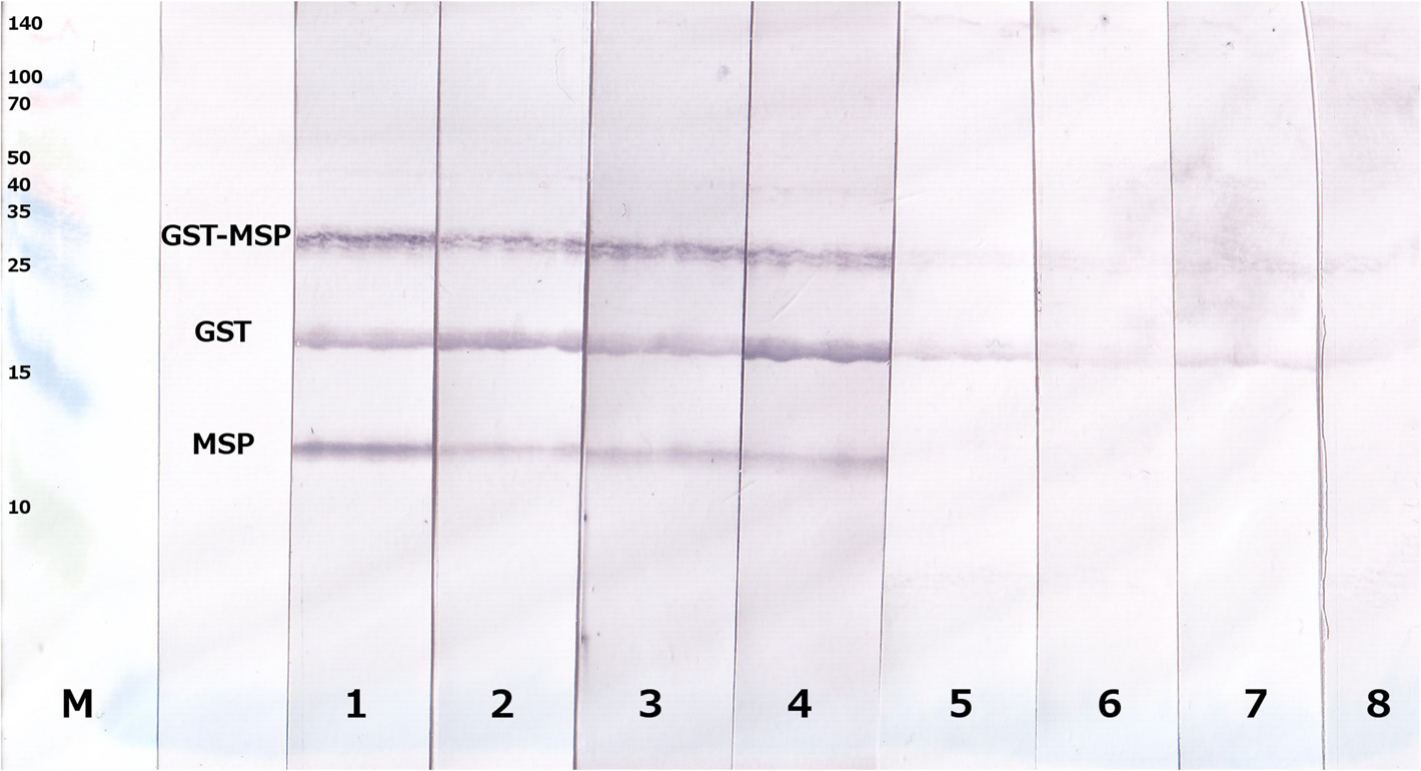
Immunoblot pattern of harbour seals. Immunoblot pattern of lungworm-positive (lanes 1–4) as well as lungworm-negative (lanes 5–8) harbour seal sera. M = Spectra™ Multicolor Broad Range Protein Ladder (Thermo Scientific)

**Fig. 3:**
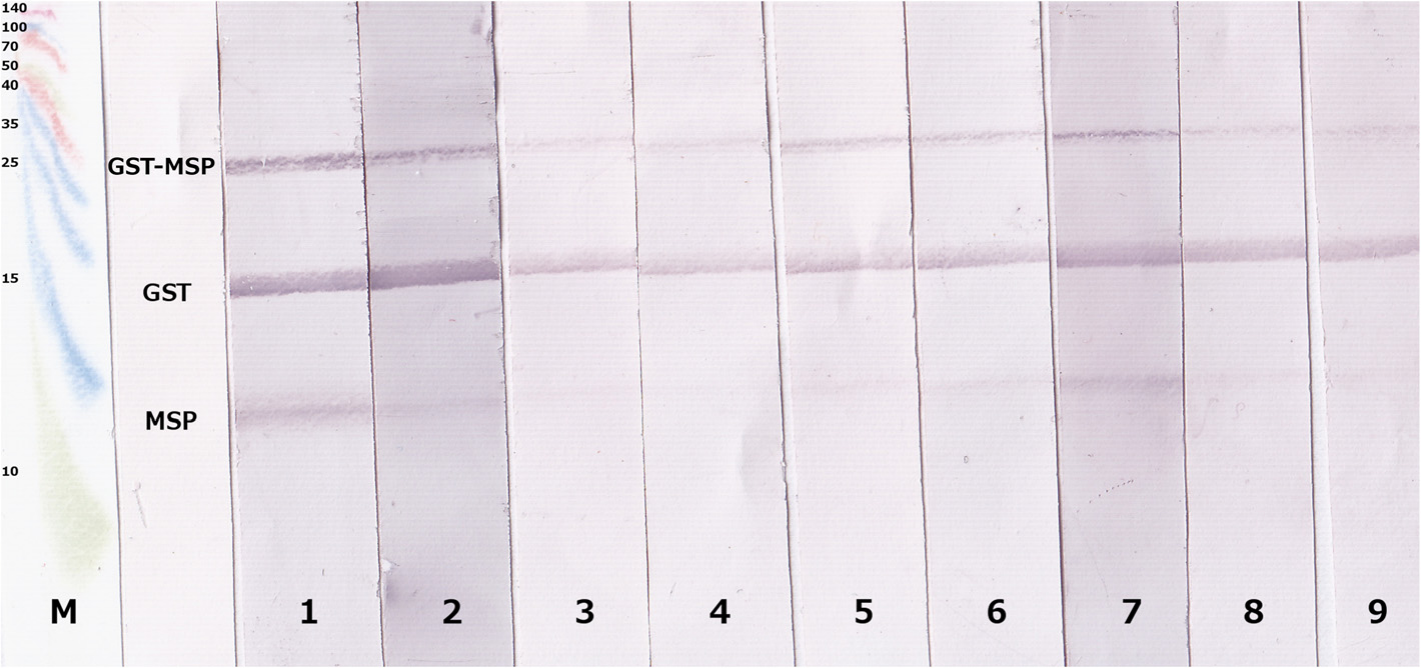
Immunoblot pattern of northern elephant and grey seals. Immunoblot pattern with serum samples of northern elephant seals, which were ELISA-positive and necropsy-positive for lungworms (lanes 1–2), ELISA-negative and necropsy-negative (lanes 3–4), ELISA-negative and necropsy-positive (lanes 5–6). Immunoblot pattern of lungworm-positive grey seals are shown in lane 7, those of lungworm-negative grey seals are shown in lanes 8–9. M = Spectra™ Multicolor Broad Range Protein Ladder (Thermo Scientific)

SDS-PAGE with a mixture GST-MSP, pure MSP and pure GST resulted in bands of about 40 kDa (GST-MSP), 26 kDa (GST) and 14 kDa, showing pure MSP. Immunoblotting with lungworm-positive harbour seal serum samples resulted in detection of all three bands, whereas lungworm-negative harbour seal sera did not detect pure MSP. However, pure GST and GST-MSP were recognised, albeit less pronounced than with positive sera (Fig. 2). Lungworm-positive and -negative sera from grey and northern elephant seals resulted basically in the same pattern except that with all negative sera a faint band appeared at the height of pure MSP (Fig. 3).

## Discussion

To date, a sensitive and individual diagnostic method for lung parasites in living seals has not been available. Clinical symptoms are only indicative of lungworm infections and faecal detection of lungworm larvae is logistically challenging in wild animals. Therefore, detection of antibodies in serum is considered the most reliable method to monitor the health status of wild living seal populations using minimally invasive sampling methods and, at the same time, to obtain individual data of seals. With the MSP-ELISA presented in this study, the desired tool has now been realised.

Bovine lungworm MSP as recombinant ELISA antigen for detection of antibodies against lungworms in cattle was first described by Schnieder [29]. A serum ELISA with 100 % specificity and sensitivity and a calculated cut-off of 0.5 OD was described using recombinant GST-MSP fusion protein as antigen for the detection of antibodies against cattle lungworms [30]. Based on the same antigen (pGEX-expressed *D. viviparus* MSP fused with *S. japonicum* GST), the ELISA presented for the detection of harbour and grey seal antibodies against the lungworms *O. circumlitus* and *P. gymnurus*, which are often found in mixed infections [8], was developed. Detection of seal antibodies by a recombinant antigen derived from the bovine lungworm *D. viviparus* demonstrates that MSP is highly conserved among nematodes [34, 35]. Binding of seal anti-MSP-antibodies to the recombinant antigen was detectable through HRP-conjugated Protein A. This protein, originally isolated from the cell wall of *Staphylococcus aureus* was used as secondary antibody as it binds particularly well to immunoglobulin G (IgG). Protein G is another IgG-binding protein, which originates from bacteria of the genus *Streptococcus*. However, Protein A was chosen over Protein G, as it has a higher affinity to dog and cat IgG. Thus, it appeared more suitable to detect the antibodies of the carnivore seals. Indeed, Protein A proved as a valuable reagent to visualise anti-MSP antibodies in seal serum. In the study by von Holtum *et al.* [30], ELISA evaluation with serum of helminth-negative calves resulted in an arithmetic mean OD of 0.17 (SD 0.083), whereas the arithmetic mean of lungworm-positive calf sera was 0.98 OD (SD 0.053). By using seal serum in a dilution of 1:100 instead of 1:40 as determined for cattle serum [30] combined with a 1:50,000 dilution of Protein A as conjugate, the harbour and grey seal-adopted MSP-ELISA resulted in arithmetic means of 0.17 OD (SD 0.082) for lungworm-negative samples and 1.775 OD (SD 0.674) for lungworm-positive samples. While negative sera of cattle and seals show an equal arithmetic mean OD value, the arithmetic mean of positive harbour and grey seal samples is considerably higher than in lungworm-positive cattle. However, ROC analysis calculated a lower cut-off (0.422 OD for the seal ELISA compared to 0.500 OD for the cattle ELISA) as positive samples showed high variations (lowest positive value: 0.467 OD; highest positive value: 2.742 OD). One reason for the high variability of seal sera is the inability to perform an experimental, controlled infection. In the study by von Holtum *et al.* [30], calves were experimentally infected with 3000–3300 infective larvae of *D. viviparus*, whereas wild seals were naturally infected, and therefore time and intensity of infection were unknown and likely variable.

Similar to the sensitivity and specificity of each 100 % [30], ROC analysis of the seal MSP-ELISA revealed 100 % specificity and 97.83 % sensitivity at the cut-off value of 0.422 OD. Out of 72 harbour and grey seal serum samples, solely one harbour seal was detected as false-negative (Fig. 1), even though a high lungworm intensity was determined during necropsy. One possible explanation might be that the immune system of the animal failed to respond properly because of weakening of the animal due to the high intensity of infection. Another explanation is that the infection was not advanced enough. In cattle, seroconversion was observed between day 26 and 41 post infection [29, 30, 36, 37], thus onset starts about one week after the beginning of patency. Onset of seroconversion after patency is a result of the nature of MSP, which is a sperm component. Thus, it is accessible for the host´s immune system only after reaching parasite adulthood and associated copulation. Therefore, the other reason for the false-negative serum sample might be that the animal was in the late prepatency or very early patency, and had not developed antibodies against MSP yet. For cattle it was stated that MSP-specific antibodies are present only in lungworm-infected animals, but not those infected with gastrointestinal nematodes [30]. This is also true for the present study: no ELISA cross-reactions with sera from harbour seals infected with either gastrointestinal nematodes or heartworms were observed.

Immunoblots with sera of positive and negative seals confirmed that the protein detected by the ELISA was MSP. Notably, all serum samples showed a positive reaction with the GST-MSP fusion protein as well as pure GST. This can be explained by cross-reactions of antibodies raised against GST of lungworms and/or other helminths with the trematode (*S. japonicum*) GST, which was used as fusion protein. After removal of GST by thrombin cleavage, it was possible to distinguish between positive and negative harbour seal sera since only lungworm-positive individuals recognised pure MSP (Fig. 2).

Immunoblot results of northern elephant seals confirmed ELISA results, which revealed solely two out of 27 lungworm-positive serum samples as antibody-positive. These two samples also recognised pure MSP in the immunoblot. However, the ELISA-negative serum samples of northern elephant seals – regardless if positive during necropsy or not – also displayed a faint pure MSP band along with MSP-GST fusion protein as well as pure GST bands. As this faint pure MSP band is also visible with sera of pre-determined negative grey seals, which were raised in human care virtually without lungworm infection risk, this band is most likely an undesired cross-reaction. As it appears in both, northern elephant and grey seals, it is presumably a cross-reaction of the AP-conjugated Protein A used as secondary antibody. Although the harbour seal immunoblot was also developed with this secondary antibody, no faint cross-reaction was visible. This can be explained by the fact that the colour reaction in the harbour seal immunoblot was stopped earlier due to an overall more intense staining reaction. Thus, the faint undesired cross-reaction did not become visible. The two northern elephant seals giving a positive ELISA result and clearly recognising pure MSP in the immunoblot were positive for faecal larvae shedding, which indicates the presence of adult lungworms. By contrast, all remaining northern elephant seals were negative for faecal larvae shedding. Indeed, Gulland *et al.* [7] observed immature stages of *O. circumlitus* in most northern elephant seals. These immature lungworm stages were mostly found in the right ventricle of the heart as opposed to the lung, as observed in harbour and grey seals. Probably, *O. circumlitus* does not find the optimal physiological environment in northern elephant seals and therefore does not develop into reproductive adult parasites. Consequently, faecal larval shedding is lacking and no MSP could be detected using the ELISA. Another explanation for the preponderance of immature parasites is that northern elephant seals are particularly susceptible to *O. circumlitus* and die before the parasites can reach sexual maturity. A mortality rate of 89 % after lungworm infection in juvenile northern elephant seals based on pulmonary arteritis and disseminated intravascular coagulation (DIC) has been described [7]. It has been speculated whether DIC is caused by larvae damaging vessel walls or by secondary bacterial infections based on release of toxic products [26]. Regardless of reasons, the use of the harbour and grey seal ELISA as a diagnostic tool in northern elephant seals cannot be recommended.

## Conclusions

The ELISA presented using recombinant MSP of the bovine lungworm *D. viviparus* as antigen allows a highly sensitive and specific determination of antibodies against lungworms in serum of harbour and grey seals during the patent phase of infection. The ELISA provides a valuable tool to reveal epidemiological dynamics of lungworm infections in wild seals. Furthermore, it may aid institutions with seals under human care with decisions on anthelminthic treatment of lungworm-infected animals.
